# Merkel Cell Carcinoma Treatment in Finland in 1986–2016—A Real-World Data Study

**DOI:** 10.3390/cancers12051224

**Published:** 2020-05-13

**Authors:** Helka Sahi, Jenny Their, Mika Gissler, Virve Koljonen

**Affiliations:** 1 Department of Dermatology, Allergology and Venerology, HUS Inflammation Center, University of Helsinki and Helsinki University Hospital, P.O. Box 160, FIN-00029 HUS Helsinki, Finland; 2 Department of Plastic Surgery, University of Helsinki and Helsinki University Hospital, FIN-00029 HUS Helsinki, Finland; jenny.their@helsinki.fi (J.T.); virve.koljonen@helsinki.fi (V.K.); 3 Finnish Institute for Health and Welfare, FIN-00271 Helsinki, Finland; mika.gissler@thl.fi; 4 Department of Neurobiology, Care Sciences and Society, Karolinska Institutet, 171 65 Solna, Stockholm, Sweden

**Keywords:** Merkel cell carcinoma, treatment, radiation therapy, immunotherapy, surgical intervention, survival, multidisciplinary communication

## Abstract

Merkel cell carcinoma (MCC) is a rare cutaneous carcinoma that has gained enormous interest since the discovery of Merkel cell polyoma virus, which is a causative oncogenic agent in the majority of MCC tumours. Increased research has focused on effective treatment options with immuno-oncology. In this study, we reviewed the real-world data on different treatments given to MCC patients in Finland in 1986–2016. We used the Finnish Cancer Registry database to find MCC patients and the Hospital Discharge Register and the Cause-of-Death Register to obtain treatment data. We identified 376 MCC patients and 33 different treatment entities and/or combinations of treatment. An increase was noted in the incidence of MCC since 2005. Therefore, the cohort was divided into two groups: the “early“ group with time of diagnosis between years 1986 and 2004 and the “late” group with time of diagnosis between 2005 and 2016. The multitude of different treatment combinations is a relatively new phenomenon; before the year 2005, only 11 treatments or treatment combinations were used for MCC patients. Our data show that combining radiation therapy with simple excision provided a survival advantage, which was, however, lost after adjustment for stage or age. Our registry study serves as a baseline treatment efficacy comparison as we move into the age of immunotherapy in MCC. Standardizing the treatment of MCC patients in Finland requires more work on awareness and multidisciplinary co-operation.

## 1. Introduction

Merkel cell carcinoma (MCC) is a rare neuroendocrine cutaneous malignancy associated with high mortality, local and regional recurrence and distant metastases [1,2]. The pathogenesis of MCC is driven by the combination of Merkel cell polyoma virus (MCPyV) infection and chronic exposure to UV radiation. The connection between MCC and MCPyV was uncovered in 2008, when a research group led by Patrick Moore discovered the presence of previously unknown polyomavirus DNA in the MCC tumour genome, later proving that viral DNA was present in the majority of MCCs [3]. Similar findings have subsequently been made in numerous studies; however, an Australian study found MCPyV to be present in only 18.3% of MCCs, giving rise to a theory that MCPyV infection is more important in MCC pathogenesis in areas of low UV exposure, whereas its significance is lower in areas of high UV exposure [4,5,6,7]. Aside from MCPyV infection and UV radiation exposure, risk factors for MCC are high age, fair skin and immunosuppression [8]. MCC most often presents in the head and neck region [9,10,11].

MCC incidence varies globally from 0.1 to 1.6 per 100,000, with the lowest incidence in Europe and the highest incidence in Australia [12,13]. The incidence has been increasing globally, with the exception of most Nordic countries [13]. MCC mainly presents in elderly patients, and rarely in patients under 50 years old, with a median age at time of diagnosis varying between 75 and 82 years [1,14,15]. Globally, MCC incidence is higher in men than in women; however, in Finland the numbers are consistently the opposite [10,13]. MCC is more common in patients with fair skin, and Caucasians present with MCC far more often than Blacks, Hispanics or Asians [13].

The treatment of MCC has seen huge developments since the advent of the sentinel lymph node biopsy (SLNB) over a decade ago. Recommended treatment for primary MCC is wide-margin excision of the primary lesion with SLNB or complete lymph node dissection (CLND) [depending on the lymph node status, followed by adjuvant radiation therapy (RT) on the primary location and/or local lymph nodes [16,17]. In metastatic MCC, chemotherapy, mainly with platinum-based therapeutics, was the only additional treatment available until 2016 [18]. Since 2016, anti-PD1/PD-L1 immunotherapies have become available and have proven effective in treatment of chemotherapy-refractory metastatic MCC [19,20]. 

MCC is a radiosensitive tumour [21]. Several studies have found that RT as an adjuvant treatment to MCC improves both locoregional control and patient overall and disease-free survival, regardless of excision margin status [14,21,22]. However, RT is not given to all MCC patients, often because the treatment can be strenuous, and the patients are usually elderly and may be suffering from comorbidities. Hypofractionated or single-fraction RT could potentially be beneficial for patients who are not eligible for longer regimens [15,23]. It has also been shown that RT effects are achieved at least partially through immune system response. RT does not only affect the targeted tumour areas, but can have a beneficial abscopal effect on malignant growth elsewhere [23,24].

The aim of this study was to examine in real-world data the different treatments given to MCC patients in Finland during 1986–2016. We also sought to examine whether adjuvant RT has a beneficial effect on patient outcome and survival in MCC in a large real-world treatment cohort of MCC patients. This data set will serve as a baseline treatment efficacy comparison as we move into the age of immunotherapy in MCC.

## 2. Results

### 2.1. Patient Demographics and Survival

**Figure 1:**
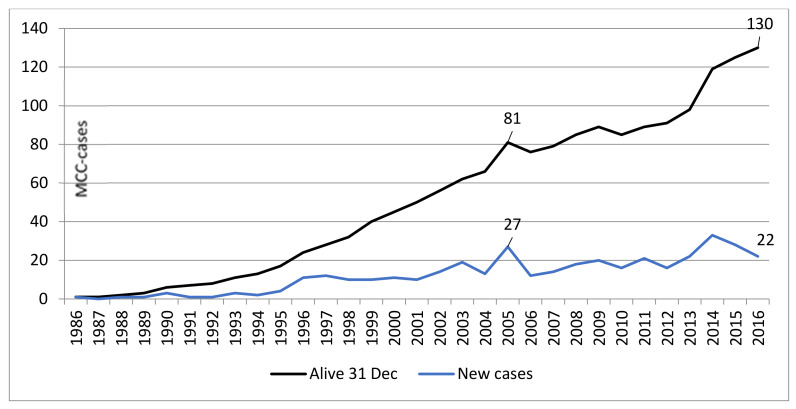
Annual incidence and prevalence of MCC in 1986–2016 in Finland.

The specific inclusion criteria resulted in 376 patients with MCC. The annual incidence varied between 1 and 33 patients (Figure 1). Figure 1 illustrates an increase in the incidence of MCC since 2005. Therefore, the cohort was divided into two groups: the “early“ group with time of diagnosis between 1986 and 2004 and the “late” group with time of diagnosis between 2005 and 2016.

In Table 1, we illustrate the detailed data of the MCC patients. Mean age at time of diagnosis was 78.7 years. There was a clear female predominance, with a male-to-female ratio of 1:1.8. Most of the tumours, 59.6%, were in the head and neck region, followed by 14.1% in the upper extremities and 12.5% in the lower extremities.

**Figure 2:**
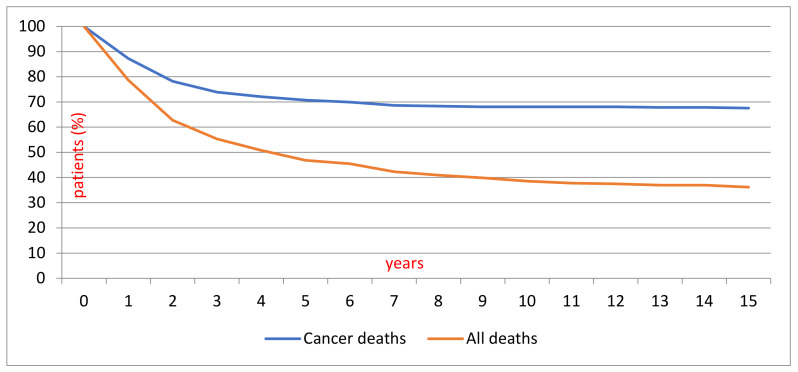
Kaplan–Meier curve depicting MCC specific deaths compared to all MCC patient deaths in 1986–2016.

By the closing date of our study on 31 December 2016, two out of three patients (65%) had died either due to MCC (27% of all) or due to other causes (38% of all). The mean overall survival was 4.2 years, and the MCC-specific survival was 1.8 years. Figure 2 shows the comparison of MCC-specific deaths and all deaths stratified by years. In the early and late cohorts, patients’ demographic data, tumour location and survival remained similar (Table 1).

### 2.2. Treatment and Outcomes

We identified altogether 33 different treatment entities or combinations of treatments (Table 2). Of the patients, 18% were recorded as having no treatment, 16% as having a single treatment and 66% as having a combination of different treatments.

In Table 3, we grouped the treatments given into three different time periods. The individual procedure codes and numbers of treated patients are listed in more detail in Table S1. The multitude of different treatment combinations is a relatively new phenomenon; before the year 2005, only 11 treatments or treatment combinations were used for MCC patients. We recorded the first SLNB in the year 2005 because the registration of the patients who had their SLNB in 2005 but were diagnosed in 2004 was not recorded in the cohort from 2005 onwards. Even excluding the SLNB, the amount of different treatments in 2005–2016 surpasses that of earlier years.

The biggest difference in the frequency of treatments was in the group “No treatment”, which declined from 34.6% to only 9.6% during the period from 2005 onwards. The MCC-specific mortality declined as well from 27.3% to 13.0% for the period 2005–2016. Any radiation therapy was given to 4.7% of the patients of the early cohort, and to 26% of the late cohort. The treatment group “Re-excision and adjuvant RT to the primary tumour” increased from 0.8% to 5.6% in the later time period (Table 3). The other treatment combinations and their stratification in separate time periods remained relatively stable.

In 2005–2016, SLNB was performed on 55 patients (22%). In 51 of these cases, re-excision was followed by SLNB, with adjuvant RT to primary tumour in 11 cases and without adjuvant RT to primary tumour in 40 cases. The MCC-specific mortality was four times higher in the group with no adjuvant RT to the primary tumour. Re-excision and SLNB was followed by CLND in only seven cases (Table 3).

Re-excision of the primary tumour was performed on 75 patients (30%) and re-excision combined with adjuvant RT to primary tumour on 14 patients (5.6%). The MCC-specific mortality was 14.5 times higher in the re-excision group that received no adjuvant RT than in the group that received adjuvant RT in addition to re-excision (Table 3). MCC-specific deaths were similar in the early and late periods (Table 1).

### 2.3. Effect of Radiation Therapy

Our data show that adjuvant RT provided a survival advantage to patients receiving simple re-excision (*p* < 0.005, Table 4). However, when standardized by stage or age, the survival advantage was lost. Due to the small number of patients in the re-excision and SLNB group compared with the re-excision and SLNB and CLND group, the difference did not reach statistical significance. MCC patients receiving RT tended to be younger, except in the re-excision and SLNB and CLND group.

## 3. Discussion

We reviewed real-world treatments of 376 patients with MCC from 1986 to 2016 in Finland. We utilized data from several national registries to obtain a representation as realistic as possible of the actual situation. The demographic data of the patients remained fairly stable over the study years. As a peculiarity to Finland [10], we once again recorded a female over-representation among MCC patients; in the years 1986–2004 the male-to-female ratio was 1:2.2 and from 2005 onwards it was 1:1.7. Despite our best efforts, this finding remains unexplained. Otherwise, our patient cohort is similar to that of previously published large series, with a mean age of 78 years at time of diagnosis, 60% of tumours located in the head and neck region and localized stage of disease [11,16,17,25]. 

Although MCC has been recognized and characterized as its own entity since 1972 [26], it was not until the discovery of Merkel cell polyoma virus in 2008 [3] that a strong interest in MCC arose. In this study, we noted an increase in the number of MCC patients in 2005 (Figure 1). In such a rare cancer as MCC, the effect of chance cannot be ruled out. In the previous literature, the increased incidence has been attributed to advances in immunohistochemistry and morphology code associated with MCC [27,28]. The growing knowledge among pathologists and clinicians translates into more MCC being diagnosed; the first thesis of our group [29] and the first article commissioned by the Finnish Medical Society [30] were published in 2004 and 2005, respectively, which may have increased the knowledge and sensitivity for MCC diagnosis.

The increase in MCC incidence shown in our study coincides with a rise in the number of treatment entities per patient and in the variety of treatment combinations. The NCCN guideline on MCC treatment was first published in 2010, followed by the European guideline in 2016 [16,17,31,32]. The latest national guideline in Finland was published after the advent of avelumab in 2017 [33]. The cornerstones of MCC treatment are re-excision, SLNB and adjuvant RT to primary tumour [16,17]. However, in 2005–2016 only 4.4% of the patients here were treated with this combination. We found that the frequency of any RT given to MCC patients in Finland increased from less than 1% to over 5% after 2005. Compared with the SEER-based results from the United States, where nearly half of the MCC patients were treated with RT [11,34], utilizing RT in MCC treatment is still less common in Finland. Relative to the years 1986–2005, there were no advances in disease-specific survival of MCC patients after 2005, despite the advent of SLNB in 2005 (Table 1). It seems that awareness of the disease or introduction of clinical guidelines has not yet translated into practice and adherence to standardized treatment protocols.

Finland faces certain challenges in the treatment of rare cancers; a small nation of just over 5.5 million people is served by five university-level tertiary centers, leading to dispersion of patients, with the physical distance to treatment facilities potentially being hundreds of kilometers. The Finnish Cancer Registry actively participates in the RARECARE initiative [35], which aims to develop the surveillance and treatment of rare cancers across Europe. In order to reach optimal treatment results while managing the costs in rare diseases, standardizing treatment protocols and establishing strong leader centers with the required expertise are of the utmost importance. Our results point to the need for better interdisciplinary communication and education within tertiary treatment centres to meet the target of standardized treatment protocols. Our findings also clearly suggest that treatment of MCC should be discussed, executed and followed by a multidisciplinary tumour board [36] because the treatment requires a wide range of specialties, including dermatologists, surgeons, radio-oncologists, medical oncologists, pathologists and radiologists.

A significant advantage in MCC-specific survival was seen with the addition of RT to primary tumour location in conjunction with simple excision. The statistical significance was, however, lost when the patients were stratified according to stage or age. Small population sizes also limited the analytical power in other treatment groups. MCC, as other neuroendocrine carcinomas, is responsive to RT and adjuvant RT is advised in the NCCN guidelines in local disease except for small low risk tumours (NCCN). Reports on the survival benefit of adjuvant RT are, however, controversial [11,37,38,39,40]. To date, the only randomized controlled study on adjuvant RT was conducted by Jouary et al. [41] on stage I MCC patients, in which RT to the tumour bed resulted in a significant decrease in local recurrence, but no significant improvement in overall survival. Subgroup analysis is oftentimes hindered in MCC studies by small sample sizes, and as such most of the clinical retrospective studies are weakened by the heterogeneity of comparable patient populations in terms of prior treatment, patient demographics, tumour characteristics and even stage of disease or target of RT. A SEER-based retrospective study postulated that the survival benefits seen in adjuvant RT are, in fact, the result of a selection bias [42].

Previous real-world studies on MCC have focused on metastatic disease [43,44]. Until recently, disseminated MCC was treated with various chemotherapeutic agents with poor results. In 2016, two separate studies proved the efficacy of immunotherapy in metastatic MCC with two molecules, avelumab and pembrolizumab [19,20]. Targeting the PD-L1/PD-1 pathway has revolutionized MCC treatment and survival and bypassed the use of cytotoxic agents in the advanced stages of MCC [17,45]. The role of RT in treatment of metastatic MCC is also undergoing a revolution. Before the advent of immunotherapy, RT was mainly seen as a palliative measure. The search to overcome refractory disease has brought with it a rise in reports on the abscopal effect in metastatic MCC [24,46]. RT modulates the tumour immunoediting process by various and partly unidentified mechanisms, such as altering tumour cell antigen presentation, increasing the infiltration of regulatory T-cells in the tumour microenvironment and staging of T-cell exhaustion with debulking the tumour mass [47,48]. Ongoing clinical trials (Clinical Trials Identifier NCT03071406, A091605) aim to shed light on the efficacy of combination treatment in advanced MCC. These might also provide new insights regarding treatment of early stage MCC.

A surprising notion arising from our large pooled data is that MCC-specific mortality seems to stabilize at seven years after the MCC diagnosis, along with the overall mortality, as seen in the Kaplan–Meier analysis (Figure 2). However, the current guidelines usually advise a five-year follow-up [16,17]. Traditionally, the survival of cancer patients has been presented at five years. Most of the adverse events occur during the first two years [49,50], with a median time to recurrence/relapse varying between 7 and 9 months [51,52]. 

Some strengths and limitations of the study warrant discussion. Coverage of the Finnish Cancer Registry is nearly 100% [53,54] of all the cancers diagnosed in Finland. Every hospital and pathology/haematology laboratory is required by legislation to submit data to the registry of all cancer patients brought to their attention. However, some individual cases might not be submitted to the Finnish Cancer Registry, and this might be especially true with unusual types of cancers like MCC, which is still poorly recognized. Likewise, the Finnish Hospital Discharge Register, maintained by the Finnish Institute of Health and Welfare, has repeatedly been shown to have completeness and accuracy levels from satisfactory to very good [55]. However, coding of the treatments in the registry is not primarily meant for research purposes or treatments; they are set as clinically indicated. Moreover, apart from data on sex, date of birth and death, we could not retrieve information on patient characteristics, including socioeconomic status or comorbidities. Prognostic tumour characteristics, such as tumour size and MCPyV status, are also beyond the scope of this study. AJCC staging was not employed in the data and staging was not updated beyond four months from diagnosis. More specific clinical findings, such as free margins and the number of positive sentinel lymph nodes, were not recorded.

All in all, our study protocol is best suited to charting the prevailing treatment patterns and general outcomes, but is unable to offer information underlying the treatment decisions such as patient comorbidities or tumour characteristics. MCC patients are elderly, and comorbidities likely explain the scarcity of invasive procedures in MCC treatment patterns. In the future, adding registry data on hospital stays and drug reimbursements could enable cost evaluation, which is needed in MCC with the advent of immunotherapy.

## 4. Materials and Methods

The study was conducted in accordance with the Declaration of Helsinki, and the protocol was approved by the Ethics Committee of The Helsinki University Hospital (Project identification code HUS/1455/2017). Permissions to identify MCC patients from the Finnish Cancer Registry, their treatment data from the National Hospital Discharge Register and data on death from the Cause-of-Death Register of Finland were obtained from the Finnish Institute for Health and Welfare and from Statistics Finland. Information from the different registers was merged through record linkages based on personal identity codes (PICs). All citizens and permanent residents in Finland have a unique PIC, which was introduced in 1964–1967. As the use of PICs enables the handling of registry data without the risk of patients being identified, patient permissions were not acquired.

In this register linkage study, the data were obtained from the Finnish Cancer Registry on all patients diagnosed with MCC from 1 January 1986 to 31 December 2016. The data included the following:

- Date of diagnosis
- Age at diagnosis
- ICD-O-3 topography
  - 440 Skin of lip, NOS, 441 Eyelid, 442 External ear, 443 Skin of other and unspecified parts of face, 444 Skin of scalp and neck, 445 Skin of trunk, 446 Skin of upper limb and shoulder, 447 Skin of lower limb and hip, 449 Skin, NOS
- Stage
  - 0 Unknown, 1 Localized, 2 Non-localized, only regional lymph node metastases, 3 Metastasized farther than to regional lymph nodes or invades adjacent tissues, 4 Non-localized, no information on extent, 5 Non-localized, also distant lymph node metastases. Stage of disease is recorded in the cancer registry files at four months after diagnosis and is not updated later.

The cohort was linked to the Cause-of-Death Register maintained by Statistics Finland. The closing date for data collection was 31 December 2016. The data included the following:

- Date of death
- Cause of death
- Deceased due to this cancer or due to other causes

The National Hospital Discharge Register maintained by the Finnish Institute of Health and Welfare was queried for the treatments given to these patients after diagnosis was assigned. The Finnish procedure coding is based on the Nordic Classification of Surgical Procedures (NCSP), which was introduced in 1997.

First, we listed all the procedures based on their frequency. In case there was no recorded treatment code and the codes were diagnostic, such as radiologic examinations, the patient was listed as having no treatment after the diagnostic biopsy date. The stratification of the procedure codes was verified by a senior author (VK) who reviewed all treatment codes and their classification case by case. The procedure codes were stratified to eight groups and to further subgroups:

1. Pre-operative RT before the re-excision of the primary tumour
2. Re-excision of primary tumour
3. SLNB
4. CLND, including partial and total parotidectomy
5. Post-operative treatment
  *i.*  adjuvant RT to the primary tumour
  *ii.*  adjuvant RT to the regional lymph nodes
  *iii.*  adjuvant cytostatic therapy
6. Therapy for progressive malignancy
  *i.*  RT of local recidive tumour
  *ii.*  cytostatic therapy of local tumour
  *iii.*  received RT of metastasis
7. Non-specified RT
8. Palliative treatment

The first SLNB was performed in 2005. Thus, the effect of RT on the survival and outcome was analyzed based on the data for 2005–2016. In this sub-cohort, we included patients whose date of diagnosis was in 2005 or later. To compare the effect of adjuvant RT in the treatment of MCC, we statistically compared re-excision, re-excision and SLNB, and CLND with and without adjuvant RT.

### Statistical Analysis

All statistical comparisons were done by using the Chi-square test, the test of relative proportion and *t*-test, where appropriate. Kaplan–Meier analysis was performed to determine overall and disease-specific survival (MCC).

All analyses were done using SAS, version 9.3 (SAS Institute Inc., Cary, NC, USA).

## 5. Conclusions

The treatments patterns of MCC in Finland are highly heterogeneous and rarely follow the international treatment guidelines. RT was rare in all disease stages. Comparison of simple excision to excision combined with adjuvant RT showed an improved survival trend, but no statistical significance was found after stratification according to disease stage. Perhaps unsurprisingly, disease-specific survival has not increased despite advances in diagnostic procedures, SLNB and the advent of treatment guidelines. This registry study serves as a baseline treatment efficacy comparison as we move into the age of immunotherapy in MCC. Importantly, the advent of immunotherapy cannot compensate for the need for proper management at the early stages of disease. Standardizing the treatment of MCC patients in Finland requires more work on awareness and multidisciplinary co-operation.

## Figures and Tables

**Table 1:** Demographic data of the 376 MCC patients treated in Finland in 1986–2016.

	Years 1986–2016	Years 1986–2004	Years 2005–2016
	N 376	N 127	N 249
**Male/female (%)**	132/244 (35/65)	40/87 (31/69)	92/157 (36/63)
**Age at diagnosis, years**			
Range	27–102	27–100	47–102
Mean (SD)	78.7 (10.6)	76.6 (12.1)	79.8 (9.6)
**Tumour location**			
440 Skin of lip, NOS	4 (1.1)	- (0.0)	4 (1.6)
441 Eyelid	11 (2.9)	4 (3.1)	7 (2.8)
442 External ear	18 (4.8)	6 (4.7)	12 (4.8)
443 Skin of other and unspecified parts of face	167 (44.4)	57 (44.9)	110 (44)
444 Skin of scalp and neck	24 (6.4)	9 (7.1)	15 (6)
445 Skin of trunk	28 (7.5)	8 (6.3)	20 (8)
446 Skin of upper limb and shoulder	53 (14.1)	17 (13.4)	36 (14)
447 Skin of lower limb and hip	47 (12.5)	16 (12.6)	31 (12.5)
449 Skin, NOS	24 (6.4)	10 (7.9)	14 (5.6)
**Stage**			
0 Unknown	158 (42.0)	42 (33)	116 (46)
1 Localized	124 (33.0)	64 (50)	60 (24)
2 Non-localised, only regional lymph node metastases	28 (7.5)	9 (7.1)	19 (7.6)
3 Metastasised farther than to regional lymph nodes or invades adjacent tissues	23 (6.1)	4 (3.1)	19 (7.6)
4 Non-localized, no information on extent	40 (10.6)	8 (6.3)	32 (12.9)
5 Non-localized, also distant lymph node metastases	3 (0.8)	- (0.0)	3 (1.2)
**Survival years, cut off 31.12.2016**			
Alive, n	130 (34.6%)	17 (13.4%)	113 (45.4%)
dead due to this cancer, n	103 (27.4%)	36 (28.3%)	67 (26.9%)
dead due to other cause, n	143 (38.0%)	74 (58.3%)	69 (27.7%)
**Mean overall survival years (SD) n**	376	127	249
diagnose date to end of surveillance/death	4.2 (4.9)	6.6 (6.7)	2.9 (2.9)
range	0-27	0–27	0–12
**Deceased due to this cancer (SD) n**	103	36	67
Mean survival, years	1.8 (2.0)	2.4 (2.6)	1.5 (1.4)
range	0–15	0–15	0.0–7
**Deceased due to other cause (SD) n**	143	74	69
Mean survival, years	4.1 (4.7)	6.1 (5.8)	2.1 (2.2)
range	0–25	0–25	0–11

**Table 2:** Different treatments and treatment combinations recorded in the study.

		Years 1986–2016	Years 1986–2004	Years 2005–2016
		N 376 (%)	N 127 (%)	N 249 (%)
No Treatment	No treatment	68 (18)	44 (34.6)	24 (9.6)
Single treatment	Re-excision of the primary tumour (Re-ex)	130 (34)	55 (43)	75 (30)
	Sentinel lymph node biopsy (SLNB)	1 (0.2)	-	1 (0.4)
	Complete lymphnode dissection (CLND)	5 (1.3)	2 (1.6)	3 (1.2)
	Pre-operative radiotherapy	1 (0.2)	-	1 (0.4)
	Adjuvant radiotherapy to the primary tumour	4 (1.1)	2 (1.6)	2 (0.8)
	Adjuvant radiotherapy to the regional lymphnodes	1 (0.2)	1 (0.8)	-
	Radiotherapy to metastasis	1 (0.2)	-	1 (0.4)
	Non-specified radiation therapy—recorded as modality	1(0.2)	-	1 (0.4)
	Non-specified radiation therapy	3 (0.8)	-	3 (1.2)
	Palliative radiotherapy	1 (0.2)	-	1 (0.4)
	Radiotherapy of metastases	2 (0.5)	-	2 (0.8)
Multiple treatments	Re-ex + SLNB	37 (9.8)	3 (2.4)	34 (13.7)
	Re-ex + CLND	50 (13.3)	16 (12.6)	34 (13.7)
	Re-ex+ adjuvant radiotherapy to the primary tumour	15 (4)	1 (0.8)	14 (5.6)
	Re-ex + chemotherapy to metastasised malignancy	1 (0.2)	-	1 (0.4)
	Re-ex + non-specified radiation therapy	6 (1.6)	1 (0.8)	5 (2)
	Re-ex + SLNB + CLND	7 (1.9)	1 (0.8)	6 (2.4)
	Re-ex + SLNB + adjuvant radiotherapy to the primary tumour	8 (2.1)	-	8 (3.2)
	Re-ex + CLND + adjuvant radiotherapy to the primary tumour	10 (2.7)	-	10 (4)
	Re-ex+ adjuvant radiotherapy to the primary tumour + radiotherapy to metastasis	1 (0.2)	-	1 (0.4)
	CLND + adjuvant radiotherapy to the regional lymphnodes	1 (0.2)	-	1 (0.4)
	CLND + radiotherapy to metastasis	1 (0.2)	-	1 (0.4)
	CLND + pre-operative chemotherapy	2 (0.5)	-	2 (0.8)
	Adjuvant radiotherapy to the primary tumour + pre-operative chemotherapy	1 (0.2)	-	1 (0.4)
	Radiotherapy to metastasis + pre-operative chemotherapy	1 (0.2)	-	1 (0.4)
	Re-ex + SLNB + non-specified radiation therapy	2 (0.5)	-	2 (0.8)
	Re-ex + SLNB + CLND + adjuvant radiotherapy to the primary tumour	1 (0.2)	-	1 (0.4)
	Re-ex + SLNB + CLND + chemotherapy to metastasized malignancy	1 (0.2)	-	1 (0.4)
	Re-ex + CLND + non-specified radiation therapy	9 (2.4)	1 (0.8)	8 (3.2)
	Re-ex + SLNB + adjuvant radiotherapy to the primary tumour + palliative radiotherapy	2 (0.5)	-	2 (0.8)
	Re-ex + CLND + adjuvant radiotherapy to the primary tumour + radiotherapy to metastasis + pre-operative chemotherapy	1 (0.2)	-	1 (0.4)
	Re-ex + CLND + adjuvant radiotherapy to the primary tumour + radiotherapy to metastasis + chemotherapy to metastasized malignancy	1 (0.2)	-	1 (0.4)

**Table 3:** Treatments and their combinations with MCC-specific survival.

Treatment	Years 1986–2016						Years 1986–2004				Years 2005–2016				
	N 376						N 127				N 249				
	N (%)	Male/Female	Mean Age Years	Death to Cancer %	Survival Years Mean	N (%)	Male/Female	Mean age years	Death to cancer %	Survival, Years Mean	N (%)	Male/Female	Mean Age Years	Death to Cancer %	Survival, Years Mean
No treatment	68 (18)	22/46	76.7	26.5	5.1	44 (34.6)	15/30	75.2	27.3	6.4	24 (9.6)	8/16	79.4	13.0	2.6
Re-excision of the primary tumour (Re-ex)	130 (34)	33/97	80.9	20.0	4.9	55 (43)	13/42	78.0	14.5	8.0	75 (30)	20/55	83.1	29.0	2.7
Sentinel lymph node biopsy (SLNB)	1 (0.2)	0/1	79.2	-	11.5						1 (0.4)	0/1	79.2	0.0	11.5
Complete lymph node dissection (CLND)	5 (1.3)	2/2	79.4	80.0	3.4	2 (1.6)	1/2	73.6	100.0	3.5	3 (1.2)	1/2	83.2	0.0	3.4
Pre-operative radiotherapy	1 (0.2)	0/1	83.9	100.0	1.8						1 (0.4)	0/1	83.9	0.0	1.8
Adjuvant radiotherapy to the primary tumour	4 (1.1)	1/3	82.3	0.0	3.7	2 (1.6)	1/1	84.2	0.0	6.5	2 (0.8)	0/2	80.4	1.0	0.8
Adjuvant radiotherapy to the regional lymph nodes	1 (0.2)	0/1	87.0	0.0	12.6	1 (0.8)	0/1	87.0	0.0	12.6	-	-/-	-	-	-
Radiotherapy to metastasis	1 (0.2)	1/0	79.7	0.0	8.1						1 (0.4)	1/0	79.7	0.0	8.1
Non-specified radiation therapy—recorded as modality	1(0.2)	0/1	86.2	100.0	1.7						1 (0.4)	0/1	86.2	0.0	1.7
Non-specified radiation therapy	3 (0.8)	1/2	89.0	66.7	1.0						3 (1.2)	1/2	89.0	1.0	1.0
Palliative radiotherapy	1 (0.2)	1/0	68.7	100.0	0.1						1 (0.4)	1/0	68.7	0.0	0.1
Radiotherapy of metastases	2 (0.5)	2/0	89.8	0.0	1.2						2 (0.8)	2/0	89.8	1.0	1.2
Re-ex + SLNB	37 (9.8)	18/19	77.8	27.0	3.6	3 (2.4)	2/1	81.1	66.7	4.2	34 (13.7)	16/18	77.5	4.0	3.5
Re-ex + CLND	50 (13.3)	22/28	78.7	34.0	3.7	16 (12.6)	7/9	75.6	56.3	3.9	34 (13.7)	15/19	80.1	9.0	3.6
Re-ex + adjuvant radiotherapy to the primary tumour	15 (4)	7/8	80.7	26.7	2.3	1 (0.8)	0/1	90.9	100.0	1.4	14 (5.6)	7/7	80.0	2.0	2.4
Re-ex + chemotherapy to metastasized malignancy	1 (0.2)	0/1	84.1	0.0	6.8						1 (0.4)	0/1	84.1	0.0	6.8
Re-ex + non-specified radiation therapy	6 (1.6)	1/5	78.7	33.3	3.7	1 (0.8)	1/0	68.7	100.0	0.3	5 (2)	0/5	80.7	2.0	4.3
Re-ex + SLNB + CLND	7 (1.9)	3/4	70.2	14.3	3.9	1 (0.8)	0/1	59.2	0.0	7.2	6 (2.4)	3/3	72.0	2.0	3.4
Re-ex + SLNB + adjuvant radiotherapy to the primary tumour	8 (2.1)	3/5	72.4	12.5	1.7						8 (3.2)	3/5	72.4	1.0	1.7
Re-ex + CLND + adjuvant radiotherapy to the primary tumour	10 (2.7)	2/8	70.2	30.0	2.7						10 (4)	2/8	70.2	0.0	2.7
Re-ex + adjuvant radiotherapy to the primary tumour + radiotherapy to metastasis	1 (0.2)	0/1	72.7	0.0	1.2						1 (0.4)	0/1	72.7	0.0	1.2
CLND + adjuvant radiotherapy to the regional lymphnodes	1 (0.2)	1/0	67.2	0.0	11.3						1 (0.4)	1/0	67.2	0.0	11.3
CLND + radiotherapy to metastasis	1 (0.2)	0/1	89.6	100.0	2.5						1 (0.4)	0/1	89.6	0.0	2.5
CLND + pre-operative chemotherapy	2 (0.5)	1/1	84.8	100.0	1.1						2 (0.8)	1/1	84.8	0.0	1.1
Adjuvant radiotherapy to the primary tumour + pre-operative chemotherapy	1 (0.2)	0/1	83.5	100.0	2.2						1 (0.4)	0/1	83.5	0.0	2.2
Radiotherapy to metastasis+ pre-operative chemotherapy	1 (0.2)	1/0	72.6	100.0	1.1						1 (0.4)	1/0	72.6	0.0	1.1
Re-ex + SLNB+ non-specified radiation therapy	2 (0.5)	0/2	83.6	0.0	3.1						2 (0.8)	0/2	83.6	1.0	3.1
Re-ex+SLNB+CLND+ adjuvant radiotherapy to the primary tumour	1 (0.2)	1/0	69.9	0.0	2.6						1 (0.4)	1/0	69.9	0.0	2.6
Re-ex + SLNB + CLND + chemotherapy to metastasized malignanacy	1 (0.2)	1/0	74.4	0.0	1.2						1 (0.4)	1/0	74.4	1.0	1.2
Re-ex + CLND + non-specified radiation therapy	9 (2.4)	5/4	75.3	66.7	2.1	1 (0.8)	1/0	49.8	100.0	2.5	8 (3.2)	4/4	78.5	2.0	2.0
Re-ex + SLNB+ adjuvant radiotherapy to the primary tumour + Palliative radiotherapy	2 (0.5)	1/1	71.6	50.0	0.8						2 (0.8)	1/1	71.6	0.0	0.8
Re-ex + CLND + adjuvant radiotherapy to the primary tumour + radiotherapy to metastasis+ pre-operative chemotherapy	1 (0.2)	1/0	76.0	0.0	1.9						1 (0.4)	1/0	76.0	0.0	1.9
Re-ex + CLND + adjuvant radiotherapy to the primary tumour + chemotherapy to metastasized malignancy	1 (0.2)	1/0	76.7	0.0	1.2						1 (0.4)	1/0	76.7	0.0	1.2

**Table 4:** Comparison of the effect of adjuvant radiation therapy to the primary tumour after excision in patients diagnosed in the year 2005 onwards.

Treatment Categories		N	Male/Female	Mean Age Years	Statistical Difference, Age, *p*	Head and Neck	Trunk	Upper Limb and Shoulder	Lower Limb and Hip	Skin, not specified	Statistical Difference, Location, *p*	Death to Cancer (%)	Statistical Difference, Death to Cancer*, p*
Excision	Re-excision of the primary tumour	75	20/55	83.1	NS	48	8	12	4	3	NS	29 (38.7)	0.005
	Re-ex + adjuvant radiotherapy to the primary tumour	14	7/7	80.0		11	1	0	2	0		0	
Excision and SLNB	Re-ex + SLNB	34	16/18	77.5	NS	18	3	5	5	3	NS	1 (2.9)	NS
	Re-ex + SLNB + adjuvant radiotherapy to the primary tumour	8	3/5	72.4		2	0	1	5	0		0	
Excision and SLNB and CLND	Re-ex + SLNB + CLND	6	3/3	72.0	NA	3	0	2	1	0	NA	1 (16.7)	NA
	Re-ex + SLNB + CLND + adjuvant radiotherapy to the primary tumour	1	1/0	69.9		0	0	1	0	0		0	
Excision and CLND	Re-ex + CLND	34	15/19	80.1	<0.001	22	1	5	3	3	NS	9 (26.5)	NS
	Re-ex + CLND + adjuvant radiotherapy to the primary tumour	10	2/8	70.2		7	1	2	0	0		0	

Re-ex = Re-excision of the primary tumour, SLNB= sentinel lymphnode biopsy, CLND = complete lymphnode dissection.

## Supplementary Materials

The following are available online at https://www.mdpi.com/2072-6694/12/5/1224/s1, Table S1: Procedures of MCC patients in 1986–2006 extracted from The National Hospital Discharge Register and listed by the procedure codes according to the Nordic Classification of Surgical Procedures.
